# The DNA resection protein CtIP promotes mammary tumorigenesis

**DOI:** 10.18632/oncotarget.8605

**Published:** 2016-04-06

**Authors:** Colleen R. Reczek, Reena Shakya, Yana Miteva, Matthias Szabolcs, Thomas Ludwig, Richard Baer

**Affiliations:** ^1^ Institute for Cancer Genetics, Department of Pathology and Cell Biology, and Herbert Irving Comprehensive Cancer Center, Columbia University Medical Center, New York, NY 10032, USA; ^2^ Current address: Department of Molecular Virology, Immunology, and Medical Genetics, Ohio State University Wexner Medical Center and Comprehensive Cancer Center, Columbus, OH 43210, USA

**Keywords:** CtIP, DNA resection, DNA break repair, chromosomal instability, tumor suppression

## Abstract

Many DNA repair factors act to suppress tumor formation by preserving genomic stability. Similarly, the CtIP protein, which interacts with the BRCA1 tumor suppressor, is also thought to have tumor suppression activity. Through its role in DNA end resection, CtIP facilitates DNA double-strand break (DSB) repair by homologous recombination (DSBR-HR) and microhomology-mediated end joining (MMEJ). In addition, however, CtIP has also been implicated in the formation of aberrant chromosomal rearrangements in an MMEJ-dependent manner, an activity that could potentially promote tumor development by increasing genome instability. To clarify whether CtIP acts *in vivo* to suppress or promote tumorigenesis, we have examined its oncogenic potential in mouse models of human breast cancer. Surprisingly, mice heterozygous for a null Ctip allele did not display an increased susceptibility to tumor formation. Moreover, mammary-specific biallelic CtIP ablation did not elicit breast tumors in a manner reminiscent of BRCA1 loss. Instead, CtIP inactivation dramatically reduced the kinetics of mammary tumorigenesis in mice bearing mammary-specific lesions of the p53 gene. Thus, unlike other repair factors, CtIP is not a tumor suppressor, but has oncogenic properties that can promote tumorigenesis, consistent with its ability to facilitate MMEJ-dependent chromosomal instability. Consequently, inhibition of CtIP-mediated MMEJ may prove effective against tumor types, such as human breast cancer, that display MMEJ-dependent chromosomal rearrangements.

## INTRODUCTION

Two major pathways exist for the repair of DNA double-strand breaks (DSBs) in eukaryotic cells: DSB repair by homologous recombination (DSBR-HR) and non-homologous end-joining (NHEJ) [1]. In addition, distinct modes of alternative end-joining (a-NHEJ or alt-EJ) repair have been identified upon analysis of cells deficient for essential components of the canonical NHEJ (c-NHEJ) pathway, such as the Ku heterodimer [2–6]. A significant fraction of these a-NHEJ events rely on short homologous sequences flanking both DSB ends to align them prior to ligation. As a consequence of this process, termed microhomology-mediated end joining (MMEJ), intervening sequences between the two microhomologies (MHs) are deleted, and a single copy of the MH is retained at the newly formed junction. Accordingly, MMEJ can be viewed as a relatively error-prone pathway of DSB repair compared to DSBR-HR or c-NHEJ.

DSBR-HR and MMEJ are both dependent on DNA resection, a nucleolytic process that converts DSB ends into 3′-single-strand DNA overhangs [1]. In mammalian cells, resection involves an initial stage in which CtIP, together with the Mre11/Rad50/Nbs1 complex, generates short single-strand DNA tails, and a subsequent stage in which these tails are elongated by the nucleolytic activities of Exo1 or Dna2 [1]. As a consequence of its role in DNA resection [7], CtIP is required for DSB repair through either the DSBR-HR or MMEJ pathways [7–12].

In addition to its role in resection, CtIP is one of several proteins that bind the C-terminal BRCT repeats of the BRCA1 tumor suppressor [13–15]. Although mice bearing a CtIP missense mutation that ablates the BRCA1/CtIP interaction are not tumor prone [16], CtIP is commonly thought to function as a tumor suppressor [17], in part based on a previous study of mice bearing a null *Ctip* allele [18]. While homozygous (*Ctip*^−/−^) animals died in early embryogenesis, heterozygous (*Ctip*^+/−^) mice were healthy, but displayed a reduced lifespan (L_50_= 625 days) relative to *Ctip*^+/+^ mice (L_50_= 780 days) [18]. On this basis, Chen *et al.* (2005) proposed that Ctip is a tumor suppressor that promotes oncogenesis upon haploid insufficiency [18]. Interestingly, however, recent studies have implicated CtIP in the formation of chromosome translocations [19, 20] and telomeric chromosomal fusions [21–23] through the error-prone MMEJ pathway of DSB repair. In principle, this aspect of CtIP function should facilitate tumorigenesis by increasing genome instability. To examine this possibility and to clarify whether CtIP can promote or suppress tumor development *in vivo*, we have examined the effects of Ctip inactivation in mouse mammary epithelial cells. Surprisingly, mammary tumors were not observed upon mammary-specific ablation of Ctip. Instead, Ctip inactivation strongly suppressed tumor formation caused by mammary-specific disruption of the p53 pathway. These results indicate that CtIP is not a tumor suppressor, but instead can promote oncogenesis, consistent with its role in the formation of chromosomal rearrangements through the MMEJ repair pathway.

## RESULTS

### CtIP heterozygosity does not increase susceptibility to tumor development

**Figure 1:**
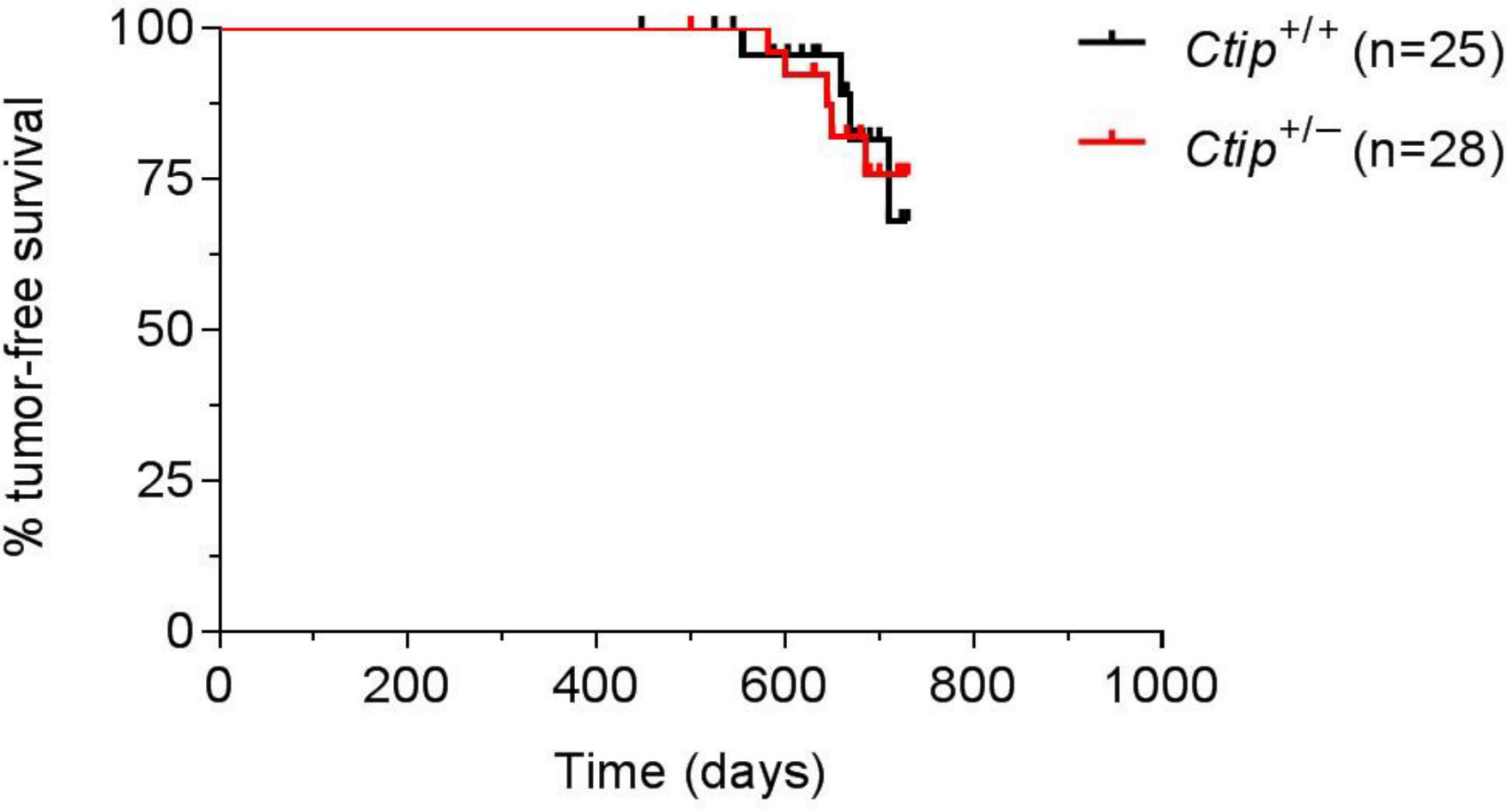
Heterozygous *Ctip*-null mice do not display increased tumorigenicity Kaplan-Meier curves of tumor-free survival are shown for wildtype *Ctip*^+/+^ (black curve; *n* = 25) and heterozygous *Ctip*^+/−^ (red curve; *n* = 28) mice monitored for tumor development for 24 months. Statistical significance (*P* = 0.9234) was estimated by the log-rank test using GraphPad Prism software; values were considered significant at *P* < 0.05.

Since CtIP is reported to promote tumor formation by haploid insufficiency [18], we monitored tumor development in cohorts of mice that do (*Ctip*^+/−^; *n* = 28) or do not (*Ctip*^+/+^; *n* = 25) harbor a single null allele of the *Ctip* gene [16]. Surprisingly, the Kaplan-Meier curves of tumor-free survival of the two cohorts were statistically indistinguishable (Figure 1), suggesting that loss of one Ctip allele did not render these animals prone to tumor development.

### CtIP does not act as a tumor suppressor in mammary epithelial cells

**Figure 2:**
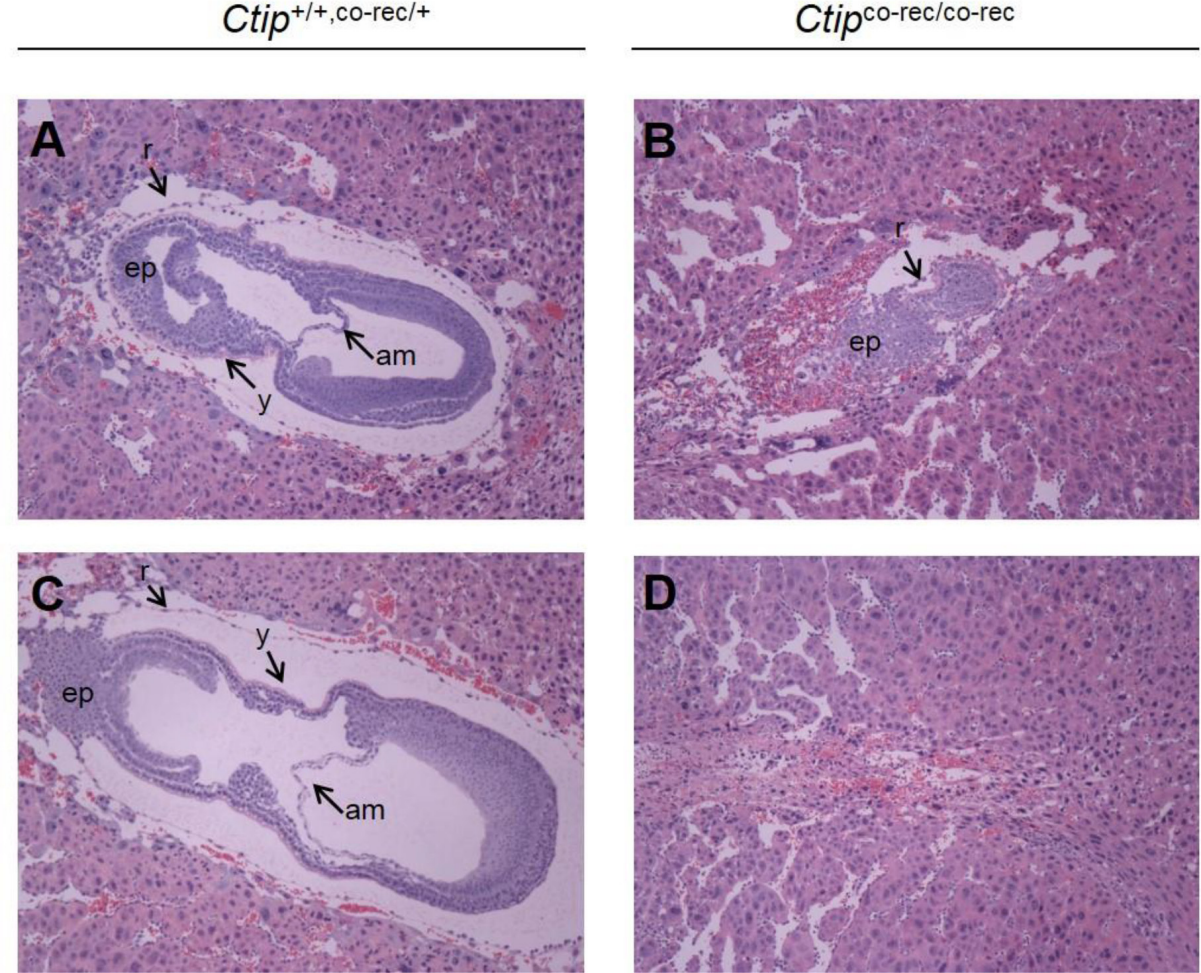
The embryonic lethality of *Ctip*^co-rec/co-rec^ embryos Hematoxylin- and eosin-stained sagittal sections of wildtype (*Ctip*^+/+^ and *Ctip*^co-rec/+^) and mutant (*Ctip*^co-rec/co-rec^) embryos at day E7.5 are shown. Wildtype (*Ctip*^+/+, co-rec/+^) post-gastrulation embryos display three distinct germ layers (**A** and **C**), while homozygous mutant *Ctip*^co-rec/co-rec^ embryos are developmentally retarded (**B** and **D**). Embryos were fixed, paraffin-embedded, sectioned, and stained with hematoxylin and eosin as described [16]. Abbreviations: am (amnion), ep (ectoplacental cone), r (Reichert's membrane), and y (yolk sac).

We next examined whether complete loss of Ctip function would elicit tumor formation. Since *Ctip*-null animals die during embryogenesis [18], we used a conditional allele (*Ctip*^co^) in which exon 2 is flanked by *loxP* recombination sites (Supplementary Figure S1D) [24]. Exon 2 contains the coding sequences for the amino-terminal 36 amino acids of Ctip. Thus, by removing the initiator methionine codon, loss of exon 2 should ablate expression of the full-length Ctip polypeptide. Although the Ctip reading frame harbors additional methionine codons downstream of exon 2, western analyses of cells harboring the Cre-recombined allele of *Ctip*^co^ (*Ctip*^co-rec^; Supplementary Figure S1E) failed to detect lower molecular-weight Ctip polypeptides (e.g., see below in Figure 5H). To confirm that the *Ctip*^co-rec^ allele is functionally null, *Ctip*^co/co^ mice were mated with animals that carry a ubiquitously expressed *Cre* transgene (*Rosa*^cre^). When *Ctip*^co-rec/+^/*Rosa*^cre/+^ progeny were intercrossed, wildtype and heterozygous pups were observed at the expected 1:2 ratio, but homozygous *Ctip*^co-rec/co-rec^ offspring were not obtained (0 of 113 viable pups). At embryonic day 7.5 (E7.5), the number of *Ctip*^co-rec/co-rec^ embryos (5 of 16 examined) approached the expected Mendelian ratio (~25%), but none of the *Ctip*^co-rec/co-rec^ embryos developed past the egg cylinder stage (Figure 2). Thus, similar to the phenotype of *Ctip*^−/−^ embryos [18], *Ctip*^co-rec/co-rec^ embryos die by the onset of gastrulation with severe growth and morphogenic defects, confirming that the Cre-recombined product (*Ctip*^co-rec^) of the *Ctip*^co^ allele is functionally null.

**Figure 3:**
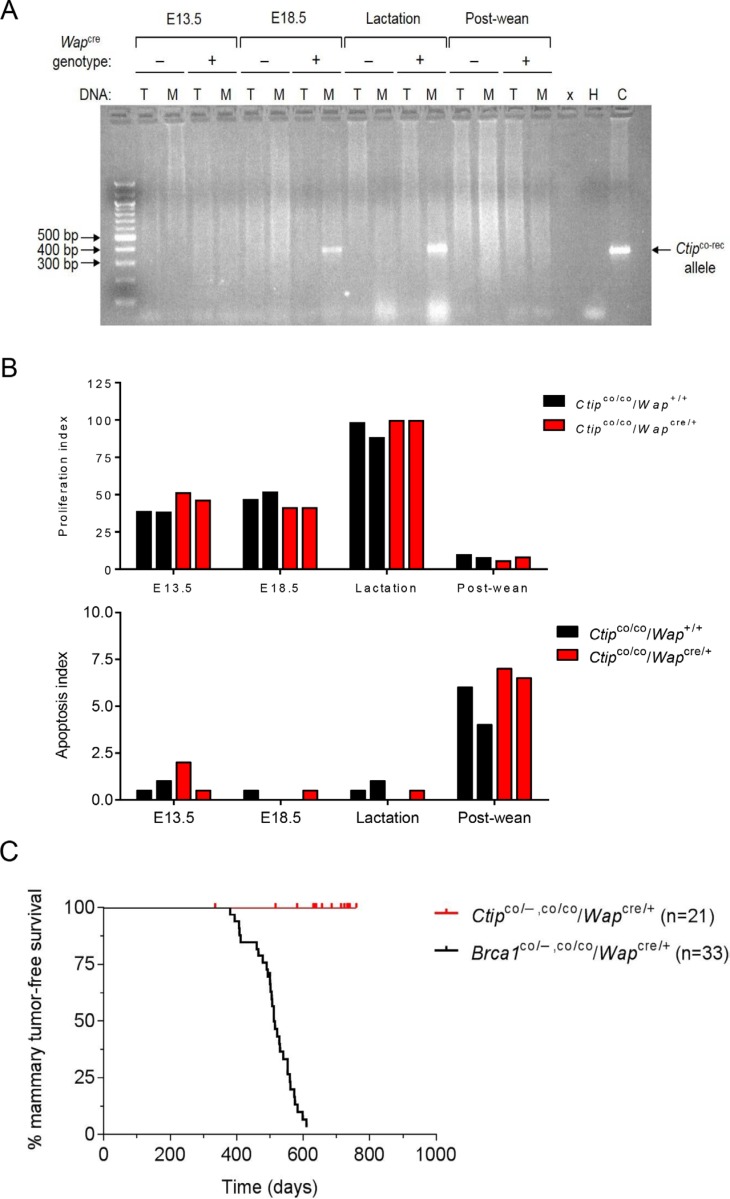
Mammary-specific Ctip inactivation does not induce breast tumors in mice (**A**) Cre-mediated *Ctip*^co^ recombination in the mammary glands of pregnant and lactating female mice. Genomic DNA was prepared from pregnant (days E13.5 and E18.5), lactating (10 days postpartum), and involuted (10 days post-wean) mammary glands of control *Ctip*^co/co^/*Wap*^+/+^ females that lack the *Wap*^cre^ transgene (−) and from experimental *Ctip*^co/co^/*Wap*^cre/+^ females that carry the *Wap*^cre^ transgene (+). PCR analysis was performed on genomic DNA from the mammary gland (M) and tail (T) of each mouse. The 350 base pair PCR product of the *Ctip*^co-rec^ allele is only observed in the mammary glands of E18.5 and lactating *Ctip*^co/co^/*Wap*^cre/+^ experimental females. Abbreviations: x (empty lane), H (water lane), and C (positive control lane). (**B**) Comparable levels of epithelial cell proliferation and apoptosis were observed in the mammary glands of experimental *Ctip*^co/co^/*Wap*^cre/+^ and control *Ctip*^co/co^/*Wap*^+/+^ mice by immunohistochemical staining for Ki67 and cleaved caspase-3, respectively. The proliferation (top bar graph) and apoptosis (bottom bar graph) indices represent the percent of Ki67- or caspase-3-positive luminal cells within the epithelial lining of tubuloalveolar structures. For each gland, a total of 200 continuous luminal epithelial cells were analyzed. The data from two mice of each genotype (experimental *Ctip*^co/co^/*Wap*^cre/+^ and control *Ctip*^co/co^/*Wap*^+/+^) are presented for each of the four developmental stages. Representative images are provided in Supplementary Figure S2. (**C**) The Kaplan-Meier curves for mammary tumor-free survival of conditional *Brca1*-null (*Brca1*^co/−^/*Wap*^cre/+^ and *Brca1*^co/co^/*Wap*^cre/+^) females (black curve; *n* = 33; T_50_ = 512 days) [26] and conditional *Ctip*-null (*Ctip*^co/−^/*Wap*^cre/+^ and *Ctip*^co/co^/*Wap*^cre/+^) females (red curve; *n* = 21; *P* < 0.0001) are compared.

To inactivate Ctip specifically in mammary epithelial cells, *Ctip*^co/co^ animals were crossed with *Ctip*^+/−^ mice that harbor *Wap*^cre^, a *Cre* transgene knocked into the whey acidic protein locus [25]. Since this is the same *Cre* transgene used to induce mammary tumors in our conditional *Brca1*-null mouse model [26], the consequences of Ctip and Brca1 inactivation can be compared in a common biological setting. The endogenous *Wap* gene is normally expressed in mammary epithelial cells during late pregnancy and lactation [27]. As expected, PCR analysis of genomic DNA identified the Cre-recombined *Ctip* allele (*Ctip*^co-rec^) in the mammary glands of late-pregnant (day E18.5) and lactating (10 days postpartum) experimental *Ctip*^co/co^/*Wap*^cre/+^ mice, but not control *Ctip*^co/co^/*Wap*^+/+^ mice (Figure 3A). Whole-mount staining revealed no apparent differences in mammary gland morphology between the experimental and control mice, and both cohorts were able to lactate and nurse their pups comparably (data not shown). Moreover, histological analyses confirmed that the proliferation (as determined by Ki67 staining) and apoptotic (staining for activated caspase-3) indices of mammary glands from experimental *Ctip*^co/co^/*Wap*^cre/+^ and control *Ctip*^co/co^/*Wap*^+/+^ mice were indistinguishable during pregnancy (days E13.5 and E18.5) and lactation (10 days postpartum) (Figure 3B and Supplementary Figure S2). In addition, mammary epithelial cell proliferation decreased and apoptosis increased to comparable levels in the involuting glands (10 days post-wean) of both the experimental and control mice (Figure 3B and Supplementary Figure S2).

To ascertain whether Ctip inactivation can elicit mammary tumors, we monitored conditional-null (*n* = 21; 7 *Ctip*^co/−^/*Wap*^cre/+^ and 14 *Ctip*^co/co^/*Wap*^cre/+^) females, each of which had been mated to induce pregnancy, Cre expression, and mammary-specific recombination of the *Ctip*^co^ allele. Unlike conditional *Brca1*-null (*Brca1*^co/−^/*Wap*^cre/+^ and *Brca1*^co/co^/*Wap*^cre/+^) females, which develop basal-like mammary tumors at a high frequency [26], all conditional-null *Ctip* animals remained mammary tumor-free over the 24-month observation period (Figure 3C), as did the control animals (*n* = 10; *Ctip*^co/+^/*Wap*^cre/+^) (data not shown). Thus, Ctip is dispensable for tumor suppression in this breast cancer model.

### Inactivation of CtIP dramatically inhibits mammary tumorigenesis in p53-deficient mice

**Figure 4:**
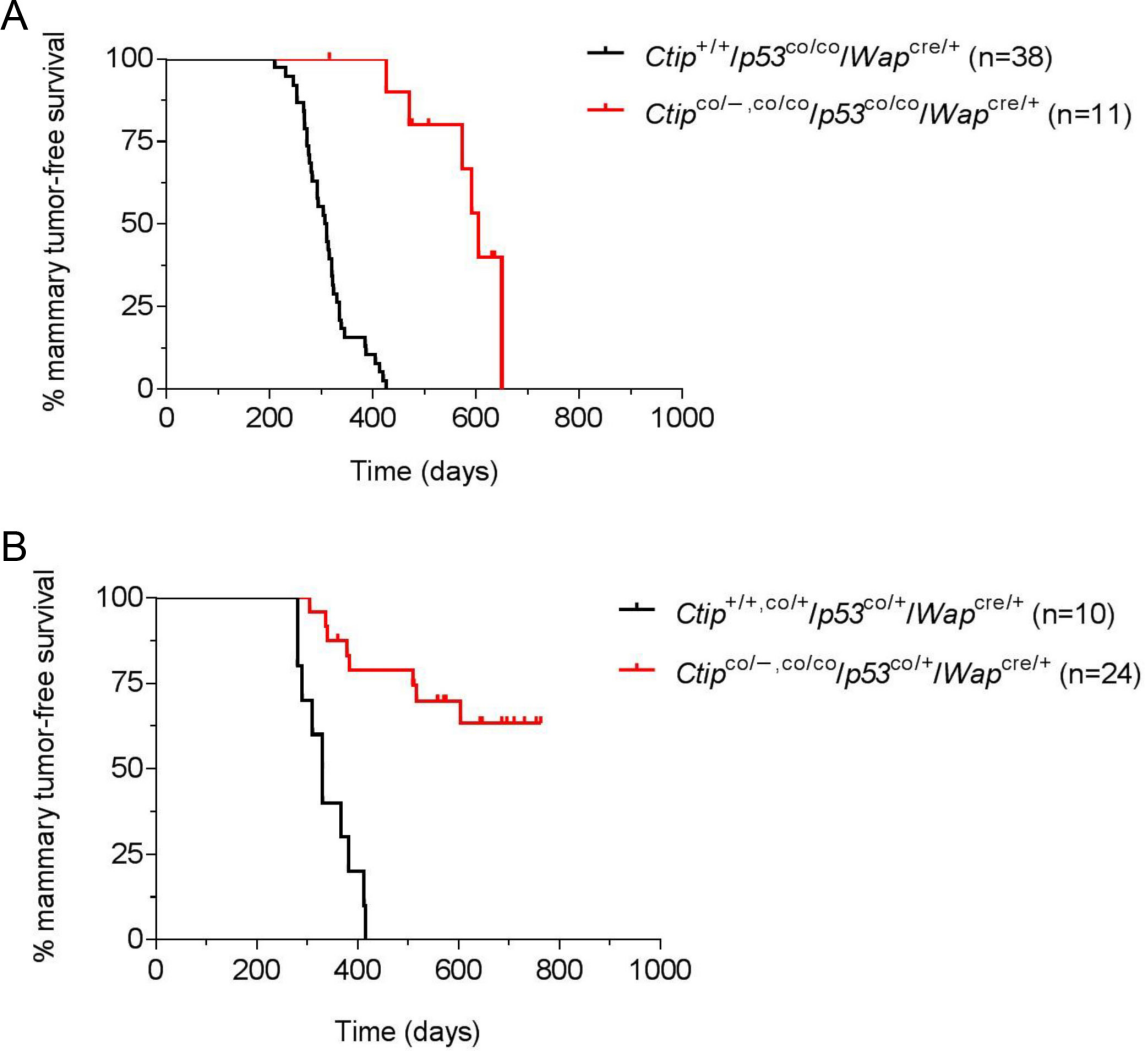
Loss of Ctip function inhibits breast cancer formation in p53-deficient mice (**A**) The Kaplan-Meier curves for mammary tumor-free survival of the control (*Ctip*^+/+^/*p53*^co/co^/*Wap*^cre/+^) females (black curve; *n* = 38; T_50_=309 days) and the experimental (*Ctip*^co/−^/*p53*^co/co^/*Wap*^cre/+^ and *Ctip*^co/co^/*p53*^co/co^/*Wap*^cre/+^) females (red curve; *n* = 11; T_50_= 605 days) are compared (*P* < 0.0001). (**B**) The Kaplan-Meier curves for mammary tumor-free survival of the control (*Ctip*^+/+^/*p53*^co/+^/*Wap*^cre/+^ and *Ctip*^co/+^/*p53*^co/+^/*Wap*^cre/+^) females (black curve; *n* = 10; T_50_= 331 days) and the experimental (*Ctip*^co/−^/*p53*^co/+^/*Wap*^cre/+^ and *Ctip*^co/co^/*p53*^co/+^/*Wap*^cre/+^) females (red curve; *n* = 24; *P* < 0.0001) are compared.

It is possible that CtIP has weak tumor suppression activity (relative to BRCA1) such that its inactivation does not appreciably affect the kinetics of mammary tumor formation in our mouse model. Thus, we also examined the effects of Ctip inactivation in a tumor-prone background caused by p53 deficiency. To disrupt p53 function, we used the conditional-null *p53*^flex7^ allele [28] (designated herein as “*p53*^co^” for clarity). Thus, in the control group (*Ctip*^+/+^/*p53*^co/co^/*Wap*^cre/+^ females), pregnancy-induced Cre expression will inactivate both *p53* alleles specifically in mammary epithelial cells. As shown in Figure 4A, these mice developed mammary carcinomas with an average latency of 309 days, consistent with previous studies [29]. If Ctip has measurable tumor suppressor activity, then concomitant inactivation of Ctip and p53 should reduce the latency of tumor formation relative to that observed upon p53 inactivation alone. In contrast, if Ctip inactivation has no influence on tumor formation, then the Kaplan-Meier mammary tumor-free survival curves resulting from co-inactivation of both p53 and Ctip and inactivation of p53 alone should statistically overlap. Instead, however, mammary tumor formation was dramatically delayed in *Ctip*^co/−, co/co^/*p53*^co/co^/*Wap*^cre/+^ females (T_50_= 605 days) relative to *Ctip*^+/+^/*p53*^co/co^/*Wap*^cre/+^ females (T_50_= 309 days) (Figure 4A). We also examined the effects of Ctip loss on mammary tumors that arise due to heterozygous p53 inactivation. As shown in Figure 4B, the control *Ctip*^+/+,co/+^/*p53*^co/+^/*Wap*^cre/+^ females developed mammary tumors with an average latency of 331 days, but tumor formation was again severely reduced by co-inactivation of Ctip (Figure 4B).

**Figure 5:**
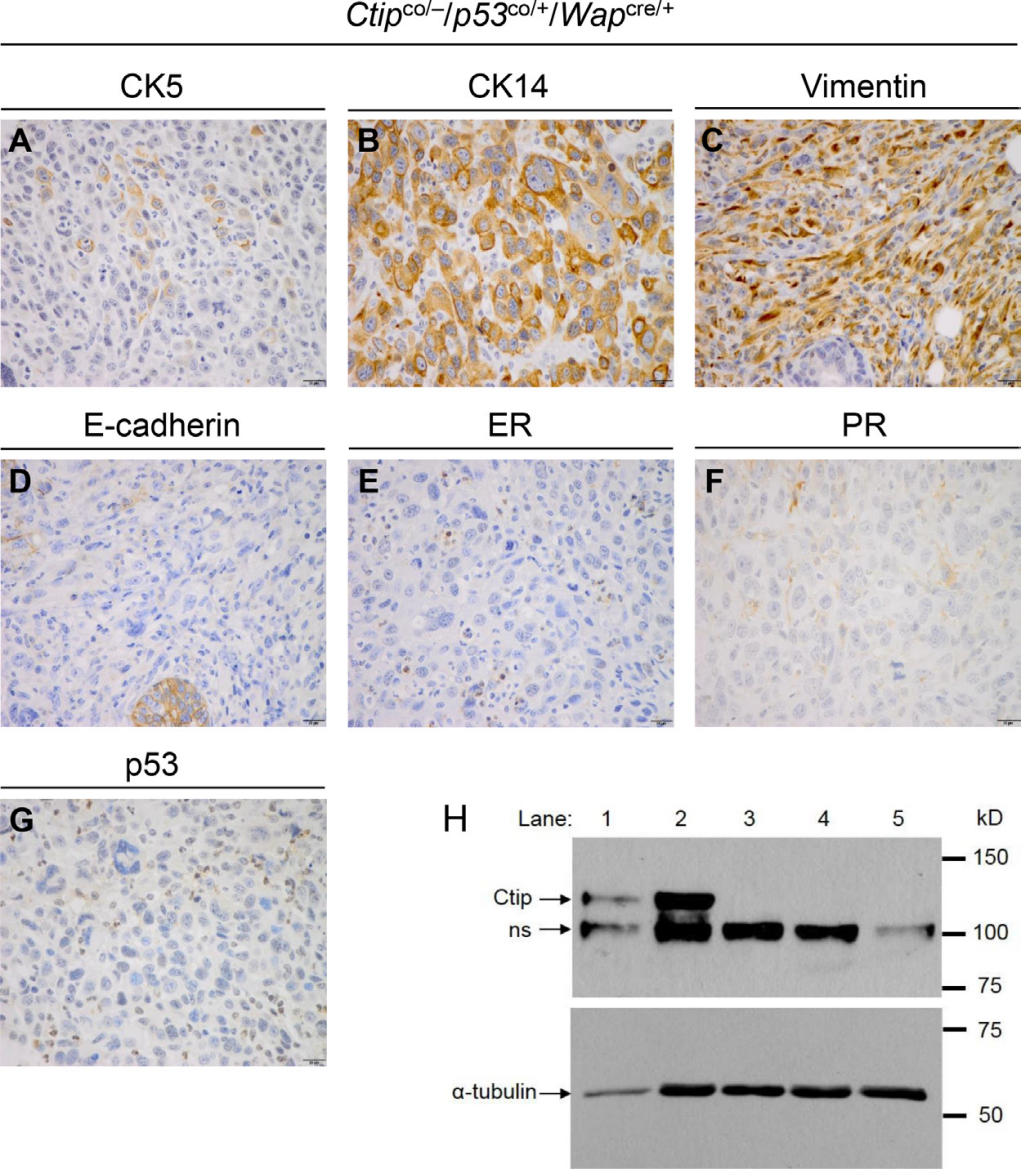
The phenotype of Ctip/p53-deficient mammary carcinomas The mammary tumors that arose with slow kinetics in the experimental Ctip/p53-mutant females (*Ctip*^co/−^/*p53*^co/+^/*Wap*^cre/+^, *Ctip*^co/co^/*p53*^co/+^/*Wap*^cre/+^, *Ctip*^co/−^/*p53*^co/co^/*Wap*^cre/+^, and *Ctip*^co/co^/*p53*^co/co^/*Wap*^cre/+^) were invasive adenocarcinomas with a predominantly solid-glandular or solid-nodular pattern. Panels (**A**–**G**) display IHC analysis of a representative *Ctip*^co/−^/*p53*^co/+^/*Wap*^cre/+^ tumor. Many of these neoplasms stained for cytoskeletal markers characteristic of basal-like breast cancer, including CK5 (6 of 10 tumors), CK14 (10 of 10 tumors), and vimentin (5 of 10 tumors) (panels A, B, and C; respectively). In addition, these tumors retained E-cadherin staining (9 of 10 tumors) (panel D), and most were estrogen receptor (ER) (7 of 10 tumors) and progesterone receptor (PR) (9 of 10 tumors) negative (panels E and F; respectively). Additionally, these tumors lacked p53 staining (panel G). (**H**) Lack of Ctip expression in the mammary tumor cells of *Ctip*^co/−^/*p53*^co/+^/*Wap*^cre/+^ mice. Whole cell lysates of mammary tumor cells from control Ctip^co/+^/*p53*^co/+^/*Wap*^cre/+^ (lanes 1 and 2) and experimental *Ctip*^co/−^/*p53*^co/+^/*Wap*^cre/+^ (lanes 3–5) mice were immunoblotted with antibodies that recognize α-tubulin or Ctip. The Ctip antiserum detects the Ctip band migrating at ~125 kD, as well as a non-specific band (“ns”) at ~100 kD.

The mammary tumors that arose with slow kinetics in the experimental Ctip/p53-mutant females (*Ctip*^co/−, co/co^/*p53*^co/+,co/co^/*Wap*^cre/+^) were invasive adenocarcinomas with a predominantly solid-glandular or solid-nodular pattern and a basal-like histopathology (Figure 5A–5F shows a representative *Ctip*^co/−^/*p53*^co/+^/*Wap*^cre/+^ tumor). As such, these tumors broadly resembled the basal-like carcinomas that develop following mammary-specific co-inactivation of Brca1 and p53 [30–32], but contrasted starkly with the sarcomatous or spindle-like breast tumors of the control p53-mutant (*Ctip*^+/+,co/+^/*p53*^co/+,co/co^/*Wap*^cre/+^) mice (data not shown). Widespread multifocal ductal carcinoma *in situ* (DCIS) was detected both adjacent and distal, and within separate mammary glands, to many of the Ctip/p53-deficient breast carcinomas, suggesting that one or more of these pre-invasive foci likely progressed to form the invasive carcinoma (data not shown). DCIS was rarely observed, however, in the mammary glands of Ctip/p53-deficient animals that remained mammary tumor free. As expected, these tumors failed to stain for p53 (Figure 5G) and Southern analyses confirmed Cre-mediated recombination of the conditional p53 allele in cultured mammary tumor cells derived from both the control (*Ctip*^co/+^/*p53*^co/+^/*Wap*^cre/+^) and experimental (*Ctip*^co/−^/*p53*^co/+^/*Wap*^cre/+^) mice (data not shown). Cytogenetic abnormalities were readily apparent in cultured mammary tumor cells derived from both the control and experimental mice, and the levels of these abnormalities were enhanced by treatment with the genotoxin mitomycin C (MMC) (Table 1). Also, as anticipated, immunoblot analysis confirmed the absence of full-length Ctip protein in mammary tumors that arose with delayed kinetics in *Ctip*^co/−^/*p53*^co/+^/*Wap*^cre/+^ females (Figure 5H, lanes 3–5), but not in the rapidly developing tumors of *Ctip*^co/+^/*p53*^co/+^/*Wap*^cre/+^ mice (lanes 1 and 2). Thus, loss of Ctip expression markedly reduces breast cancer formation in p53-deficient mice.

**Table 1:** Spontaneous and MMC-induced chromosomal aberrations in mouse mammary tumor cells with different Ctip genotypes

Mammary tumor cell line	Metaphases analyzed	MMC treatment	Metaphase aberrations, %	Chr/Cht: breaks and gaps	Exchange/Other
Control (1)	20	–	35	6	0
	30	+	70	32	0
Control (2)	20	–	30	5	0
	30	+	67	29	2
Experimental (1)	20	–	45	9	0
	30	+	87	51	2
Experimental (2)	20	–	30	8	0
	30	+	87	57	3

The percentage of metaphases containing one or more chromosomal aberrations, as well as a breakdown of aberration type, is shown for two control (*Ctip*^co/+^/*p53*^co/+^/*Wap*^cre/+^) and two experimental (*Ctip*^co/−^/*p53*^co/+^/*Wap*^cre/+^) mammary tumor cell lines in both the absence (−) and presence (+) of MMC treatment. Abbreviations: MMC (mitomycin C), Chr (chromosome), and Cht (chromatid).

### CtIP loss also inhibits mammary tumors induced by a dominant-negative p53 allele

**Figure 6:**
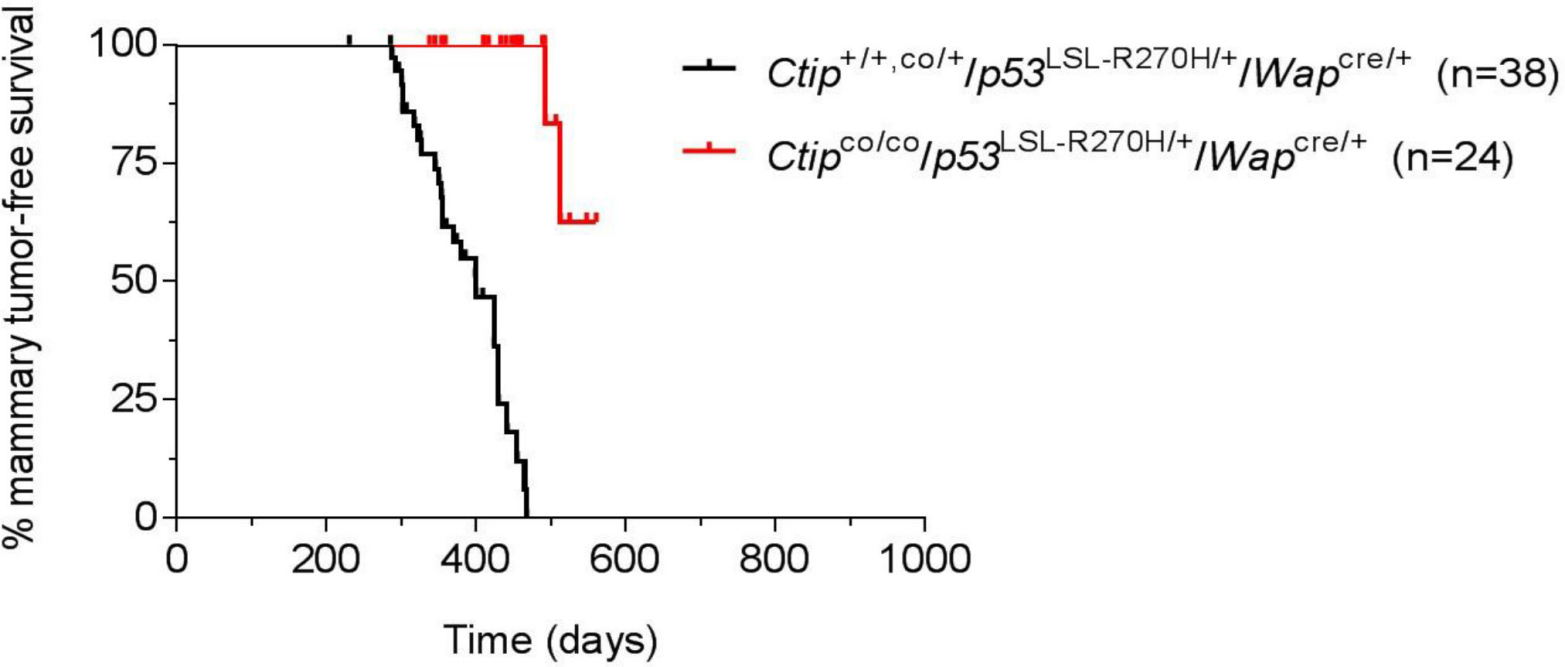
Loss of Ctip inhibits breast cancer induction by a dominant-negative p53 mutation The Kaplan-Meier curves for mammary tumor-free survival of the control (*Ctip*^+/+^/*p53*^LSL-R270H/+^/*Wap*^cre/+^ and *Ctip*^co/+^/*p53*^LSL-R270H/+^/*Wap*^cre/+^) females (black curve; *n* = 38) and the experimental (*Ctip*^co/co^/*p53*^LSL-R270H/+^/*Wap*^cre/+^) females (red curve; *n* = 24) are compared (*P* < 0.0001).

Since the p53 lesions associated with human cancer are mostly dominant-negative missense mutations, we also examined the effects of Ctip inactivation in mice bearing the conditional *p53*^LSL-R270H^ allele, which encodes murine *p53* with a mutation corresponding to the human R273H hotspot mutation often associated with breast cancer [29, 33]. The *p53*^LSL-R270H^ allele was designed such that p53^R270H^ expression is blocked until removal of a Lox-STOP-Lox cassette by Cre recombinase [33]. To examine the effect of Ctip inactivation in this tumor-prone setting, we generated cohorts of experimental (*Ctip*^co/co^/*p53*^LSL-R270H/+^/*Wap*^cre/+^) and control (*Ctip*^+/+, co/+^/*p53*^LSL-R270H/+^/*Wap*^cre/+^) females. As shown in Figure 6, mammary tumors developed in the control *Ctip*^+/+,co/+^/*p53*^LSL-R270H/+^/*Wap*^cre/+^ females with an average latency of 400 days, consistent with previous studies [29, 32]. Significantly, however, Ctip inactivation markedly reduced both the incidence and kinetics of tumor development in the experimental *Ctip*^co/co^/*p53*^LSL-R270H/+^/*Wap*^cre/+^ females (Figure 6). Thus, Ctip inactivation inhibits the formation of mammary tumors caused by either homozygous or heterozygous *p53* gene inactivation (Figure 4) or by a dominant-negative p53 missense mutation frequently observed in human breast cancer (Figure 6).

## DISCUSSION

The CtIP protein is commonly thought to function as a tumor suppressor [17], a view predicated in part on its interaction with BRCA1 [13]. Indeed, Chen *et al.* (2005) proposed that Ctip promotes tumor formation by haploid insufficiency based on a reduced lifespan of *Ctip*^+/−^ mice (L_50_= 625 days) relative to *Ctip*^+/+^ mice (L_50_= 780 days) [18]. Surprisingly, however, we observed no difference in either lifespan or the kinetics of tumor formation in wildtype (*Ctip*^+/+^) and heterozygous (*Ctip*^+/−^) mice (Figure 1), suggesting that loss of one *Ctip* allele does not impart a heightened susceptibility to cancer. Although we are not certain why our results differ from those reported by Chen *et al.* (2005), several points are worth noting. First, Chen *et al.* (2005) compared overall survival of *Ctip*^+/+^ and *Ctip*^+/−^ mice, not actual tumor-free survival. Also, no statistical analysis was provided to support their contention that the reported lifespan difference between *Ctip*^+/+^ and *Ctip*^+/−^ mice is biologically meaningful. Furthermore, while Chen *et al.* (2005) observed tumors in *Ctip*^+/−^ mice, histopathological analysis of moribund animals in the control *Ctip*^+/+^ cohort was not addressed. Lastly, the genetic background of mice is known to affect tumor susceptibility. Our experimental (*Ctip*^+/−^) and control (*Ctip*^+/+^) cohorts were comprised of littermates with a mixed genetic background (C57BL/6J x 129/Sv (B6;129)). Although the background of the mice used by Chen *et al.* (2005) and the relationship between their cohorts were not specified, the tumor spectrum (predominantly lymphomas) of their *Ctip*^+/−^ mice closely resembles that of aging C57BL/6-inbred and B6;129 non-inbred strains [34]. In light of these factors, we conclude that loss of one Ctip allele does not render mice prone to tumor development (Figure 1). Moreover, through analysis of *Ctip* conditional-null mice, we further show that complete loss of Ctip function does not elicit tumor formation in mammary epithelial cells (Figure 3C). Together, these data suggest that Ctip, unlike Brca1, is dispensable for tumor suppression *in vivo*.

To confirm that CtIP lacks tumor suppression activity, we also examined the effect of Ctip ablation on mammary tumors induced by p53 deficiency. Surprisingly, in this setting Ctip inactivation dramatically reduced the kinetics of mammary tumor formation (Figures 4 and 6). Since *Ctip*-null mice undergo embryonic lethality ([18] and Figure 2), it is conceivable that Ctip loss inhibits tumor development by reducing the viability of precancerous mammary epithelial cells. Nevertheless, mammary-specific inactivation of either the *Brca1*, *Bard1*, or *Brca2* gene readily induces mammary tumors [25, 26, 30–32, 35] despite the fact that mice lacking expression of these genes also die during early embryogenesis [36–41]. Moreover, no morphological defects in mammary gland development or changes in mammary epithelial cell proliferation or apoptosis were observed upon conditional Ctip inactivation (Figure 3B and Supplementary Figure S2). Also, the malignant cells of mammary tumors that arose with delayed kinetics in *Ctip*^co/−^/*p53*^co/+^/*Wap*^cre/+^ females were clearly viable, both *in vivo* and *in vitro*, despite the complete absence of Ctip expression (Figure 5H). Therefore, in light of these observations, we must consider the possibility that wildtype Ctip can actually facilitate mammary tumorigenesis in p53-deficient mice. Although at first glance an oncogenic function for CtIP seems counterintuitive, it may reflect the ability of CtIP to promote the formation of chromosome translocations [19, 20] and/or telomeric chromosomal fusions [21–23] through microhomology-mediated end joining (MMEJ). In addition, since CtIP can enhance the nuclease activity of Mre11 [7], it may also facilitate the chromosomal rearrangements that arise upon Mre11-dependent degradation of stalled replication forks [42–44].

Most human carcinoma cells harbor numerous chromosomal rearrangements, at least some of which are likely to represent genetic lesions that drive malignant development [45]. In experimental systems, DNA double-strand breaks (DSBs) dramatically increase the frequency of chromosome translocations, suggesting that translocations arise when DSBs at distal genomic sites are resolved by endogenous DSB repair pathways [46]. Interestingly, the junctions of tumor-associated chromosomal rearrangements frequently exhibit microhomologies (MHs) indicative of MMEJ repair [2, 4, 5, 47–50]. For example, genome-wide sequence analysis of twenty-four breast tumors uncovered MHs at the junctions of most (~65%) chromosomal rearrangements [51], suggesting that MMEJ is a major contributor to genomic instability in human breast cancer. In addition, early studies also observed junctional MHs in the oncogenic chromosome translocations that arise in the B cell lymphomas of c-NHEJ-deficient mice [52, 53]. Thus, chromosomal rearrangements may arise during mammary tumorigenesis when DSBs are repaired illegitimately through the error-prone MMEJ pathway rather than through preferred modes of repair, such as DSBR-HR or c-NHEJ (Supplementary Figure S3). This notion is supported by evidence that inactivation of CtIP, which is required for MMEJ [8–10], suppresses the formation of MH-bearing chromosome translocations in embryonic stem cells [19].

A substantial body of evidence has implicated MMEJ as the major DSB repair pathway that generates chromosomal translocations in murine cells [19, 48, 52, 53]. Interestingly, however, it was recently proposed that the mechanisms of chromosomal translocation vary between species, such that translocations are predominantly formed by alternative end-joining (a-NHEJ or alt-EJ) pathways such as MMEJ in mouse cells and by c-NHEJ in human cells [54]. Nevertheless, subsequent studies have shown that a-NHEJ is a major source of chromosome translocations in human cells [55] and that CtIP in particular can promote the formation of chromosomal rearrangements in human cells [20]. Conceivably, the repair pathways that generate translocations may differ depending on tissue type rather than species. Indeed, genome-wide sequencing studies of human cancer indicate that the mechanisms underlying chromosomal instability are likely to be diverse and may vary with respect to tumor type [56]. For example, while MHs are found at the junctions of most chromosomal rearrangements in human breast [51] and pancreatic [57] tumors, junctional MHs are seldom associated with those of human prostate cancer [58]. Interestingly, recent studies have shown that Polθ, a low fidelity DNA polymerase implicated in a subset of MMEJ repair events [59–63], is frequently overexpressed in several human tumor types, including breast cancer and serous ovarian cancer [63–65]. Thus, the genomic instability that characterizes, and presumably drives, breast carcinogenesis may be especially dependent on the MMEJ repair pathway. If so, then inhibition of CtIP function may prove an effective means to prevent or treat tumor types, such as human breast cancer, that display MMEJ-dependent chromosomal instability.

## MATERIALS AND METHODS

### Mouse strains

The *Ctip*^−^, *Wap*^cre^, and conditional-null *p53*^flex7^ (*p53*^co^) alleles were described previously [16, 25, 28] and the *p53*^LSL-R270H^ mouse strain (number 01XM3) [29] was obtained from the NCI-Frederick Mouse Repository. Animals were maintained on a mixed genetic background consisting of 129/Sv and C57BL/6J. In studies of tumor formation, the experimental and control cohorts were comprised of littermates. Animal care was performed in accordance with the National Institutes of Health guidelines in the AAALAC-accredited animal facility at Columbia University.

### Production of the conditional-null *Ctip*^co^ allele

The conditional-null *Ctip*^co-neo^ targeting construct consists of a 7.7 kb genomic DNA fragment containing exon 1 and exon 2 of Ctip (Supplementary Figure S1B) [24]. The construct was generated by flanking exon 2, which encodes the N-terminal 36 amino acids of Ctip, with two *loxP* recombination signals. One *loxP* signal was introduced upstream of the transcriptional initiation site in the *Nhe*I restriction site of intron 1, and the second *loxP* signal was inserted, along with a PGK-neomycin resistance cassette flanked by *FRT* (Flp recombinase target) signals, into the *Eco*RV restriction site of intron 2. In addition, a herpes simplex virus type 1 thymidine kinase (HSV-TK) negative selection gene cassette was included to select against random integration. The conditional-null targeting construct (Supplementary Figure S1B) was electroporated into 129/Sv embryonic stem (ES) cells as described previously [16], and two independent neomycin-resistant ES clones harboring the *Ctip*^co-neo^ knock-in allele (Supplementary Figure S1C) were identified and injected into C57BL/6J blastocysts. Germline-transformed *Ctip*^co-neo/+^ heterozygous mice were mated with Flpe-expressing mice to produce offspring in which the *FRT*-flanked neomycin cassette had been excised, thereby converting the *Ctip*^co-neo^ allele into the desired *Ctip*^co^ allele (Supplementary Figure S1D).

### PCR and immunohistochemical analyses of mammary glands

To assess Cre-dependent recombination of the *Ctip*^co^ allele (Supplementary Figure S1D), the recombined *Ctip*^co-rec^ allele (Supplementary Figure S1E) was amplified from genomic DNA of mouse tails and mammary glands. The PCR reactions were conducted using *Taq* DNA Polymerase (Invitrogen) in 1X PCR buffer (Invitrogen), 50 mM MgCl_2_, 10 mM dNTPs, 100% DMSO, 1 μl of genomic DNA, and 10 mM of the forward (5′-GGGCTCAGTTTCTGGGTGCT) and reverse (5′-TTGCAGAGAACCAAAGTTCAGC) primers. The 350 base pair PCR fragment was amplified under the following conditions: 94°C for 3 min (1 cycle); 94°C for 30 sec, 58°C for 30 sec, 72°C for 30 sec (40 cycles); 72°C for 3 min (1 cycle); 4°C hold. For immunohistochemical analyses, sections of mammary gland #4 from pregnant (days E13.5 and E18.5), lactating (10 days postpartum), and weaned mother (10 days post-wean) mice were stained using the immunoperoxidase technique with antibodies for the proliferation marker Ki67 (clone MIB-1, Dako) and the apoptosis marker cleaved caspase-3 (Asp175 antibody, Cell Signaling Technology). Proliferation and apoptosis indices were then calculated as the percentage of Ki67- or caspase-3-positive luminal cells within the epithelial lining of tubuloalveolar structures. For each gland, a total of 200 continuous luminal epithelial cells were analyzed.

### Tumor monitoring, histopathology, tumor cell culturing, and cytogenetic analysis

Tumor monitoring and histological analyses were conducted as described previously [16]. Mammary tumor specimens were stained with antibodies against E-cadherin (BD Pharmingen), vimentin (Research Diagnostics Inc.), CK5 and CK14 (Covance), ERα (Santa Cruz), PR (Affinity BioReagents), and p53 (Novocastra NCL-p53-CM5p polyclonal). In addition, decidua dissected at embryonic day 7.5 were fixed, paraffin-embedded, sectioned, and stained with hematoxylin and eosin as described [16]. To establish primary mammary tumor cell lines, mice were sacrificed two weeks after mammary neoplasms were detected by palpation. Pieces of tumor tissue were then diced, trypsinized, and passed through a needle several times, and mammary tumor cells were cultured for at least six passages in DMEM (Cellgro) with 10% heat-inactivated FBS (Tissue Culture Biologicals), 100 μg/ml penicillin/streptomycin, 2 mM L-glutamine, and 1.25 μg/ml Plasmocin (InvivoGen) at 37°C in 5% CO_2_/95% humidity. For analyses of Ctip protein expression, total cell extracts prepared from several control heterozygous p53 conditional-null and experimental Ctip/p53 double conditional-null mammary tumor cell lines were fractionated by PAGE and immunoblotted with antibodies specific for CtIP [14] or α-tubulin (DM1A; EMD Millipore). For cytogenetic analyses, metaphase spreads were prepared from several mammary tumor cells lines derived from control (*Ctip*^co/+^/*p53*^co/+^/*Wap*^cre/+^) and experimental (*Ctip*^co/−^/*p53*^co/+^/*Wap*^cre/+^) mice. Briefly, after treatment with or without mitomycin C (40 ng/ml) for 16 hours, the cells were cultured for 2 hours with 0.05 μg/ml KaryoMAX Colcemid solution (Gibco), followed by a 20 minute exposure to a 0.38% KCl (w/v) hypotonic solution, and methanol/acetic acid (3:1) fixation. Fixed cell suspensions were dropped onto glass slides and stained with Giemsa (Gibco).

### Statistical analysis

Mouse tumor-free survival is represented with Kaplan-Meier curves, and the significance was estimated with the log-rank test using GraphPad Prism software (version 6). Values were considered statistically significant at *P* < 0.05.

## SUPPLEMENTARY MATERIALS FIGURES


