# Supplementary material for: The DNA resection protein CtIP promotes mammary tumorigenesis
Source: Oncotarget. 2016 Apr 6;7(22):32172–83. doi: 10.18632/oncotarget.8605 (PMC5078005; doi:10.18632/oncotarget.8605)
Supplement: Supplementary file 1 [file oncotarget-07-32172-s001.pdf]

The DNA resection protein CtIP promotes mammary tumorigenesis

Supplementary Materials

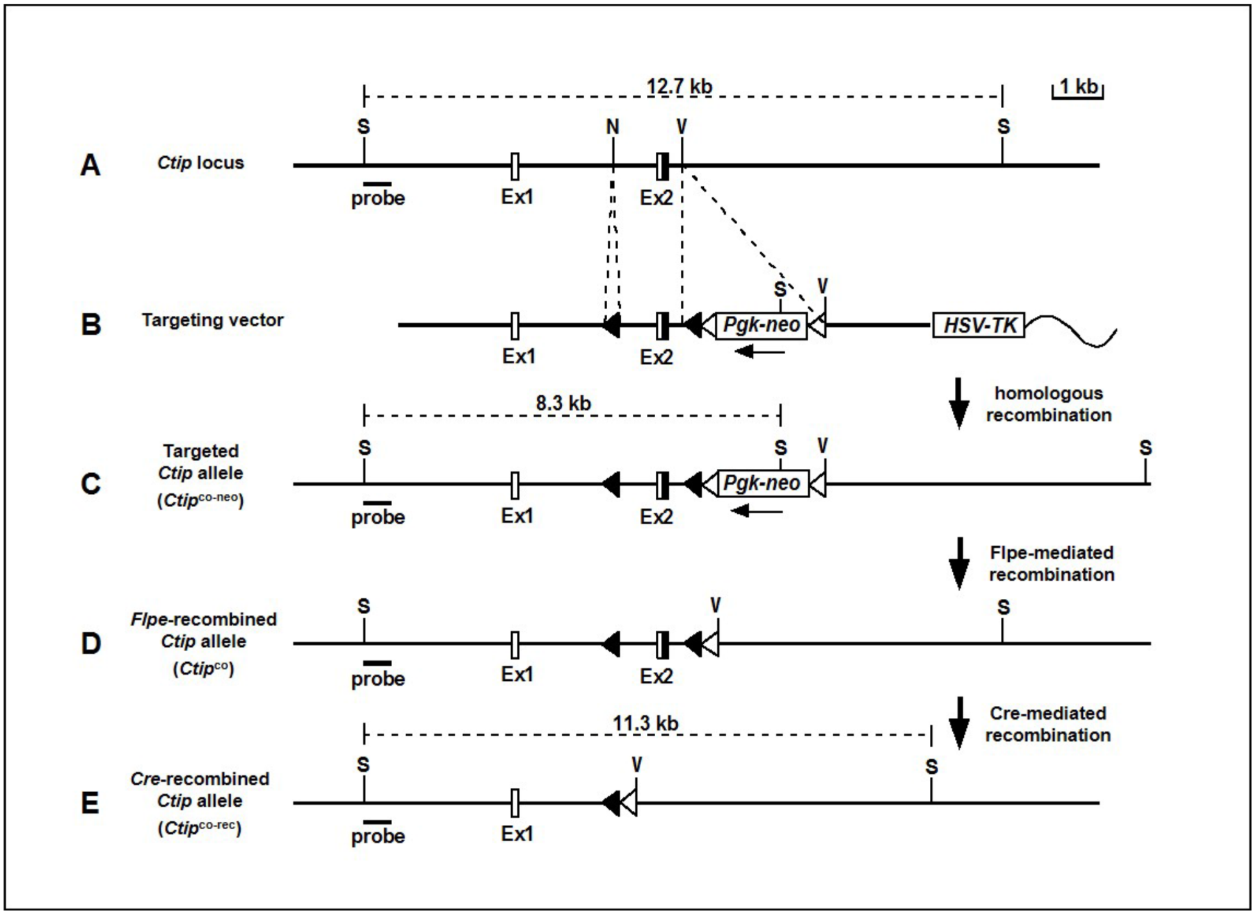

Supplementary Figure S1: Design of the conditional-null *Ctip*^{co} allele. The wildtype *Ctip* locus encompassing exons 1 and 2 is shown (**A**), along with the targeting vector (**B**), and maps of the *Ctip* locus after homologous recombination (**C**, *Ctip*$^{co\text{-}neo}$), Flpe-mediated recombination (**D**, *Ctip*^{co}), and Cre-mediated recombination (**E**, *Ctip*$^{co\text{-}rec}$). Note that the Flpe-recombined locus illustrated in D corresponds to the conditional-null *Ctip*^{co} allele established in mice. The coding sequences of *Ctip* exons are represented by filled rectangles while non-coding sequences are depicted by open rectangles. For the targeting vector, a single *loxP* recombination signal (filled triangle) was inserted into intron 1, while a second *loxP* signal, together with a PGK promoter-driven neomycin expression cassette flanked by *FRT* signals (open triangles), were inserted into intron 2. An HSV thymidine kinase (HSV-TK) gene cassette was included in the targeting vector for negative selection. The wavy line represents plasmid sequences of the targeting vector. Restriction sites are: *NheI* (N), *EcoRV* (V), and *SpeI* (S). The Ctip probe used for Southern analysis to identify properly targeted *Ctip*$^{co\text{-}neo/+}$ ES clones is shown, as are the sizes of the *SpeI* fragments recognized by this probe.

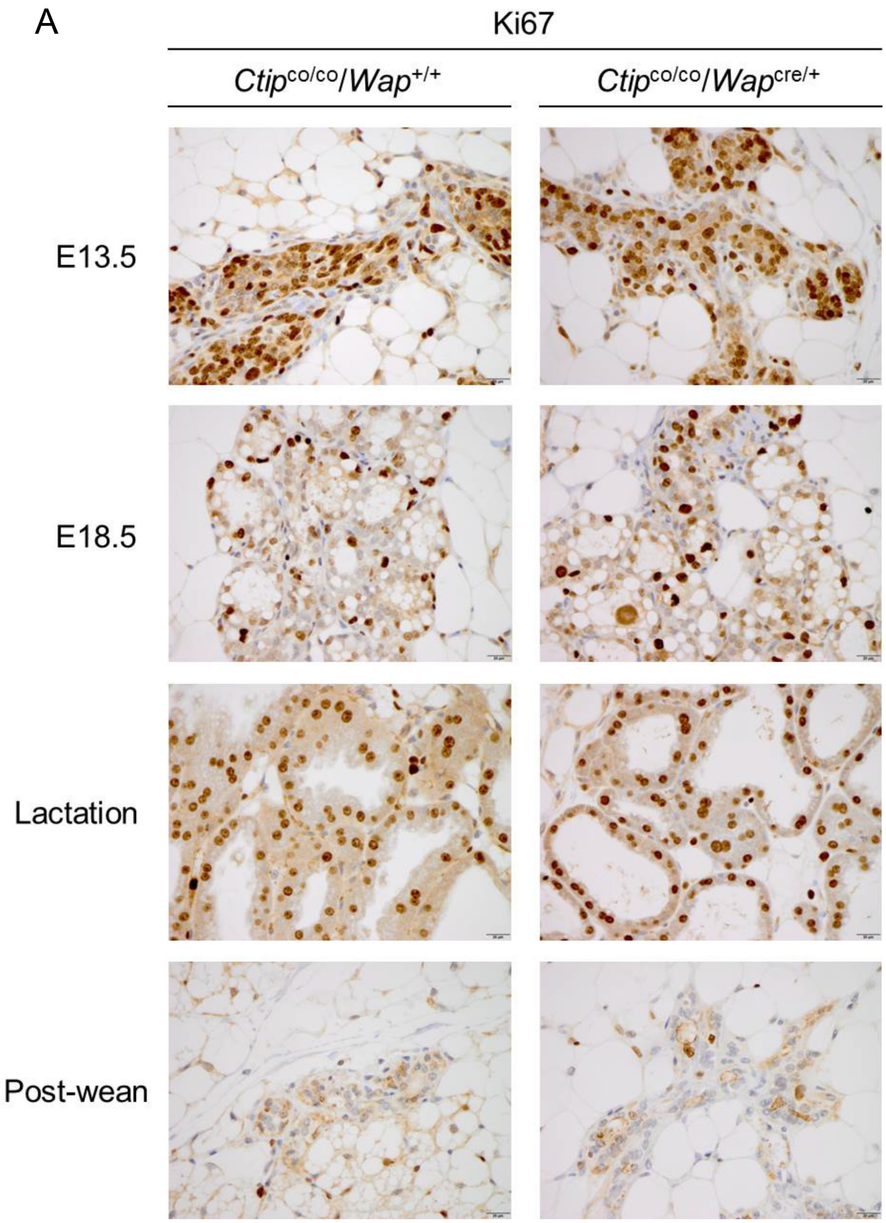
A
Ki67
Ctipco/co/Wap+/+
Ctipco/co/Wapcre/+
E13.5
E18.5
Lactation
Post-wean

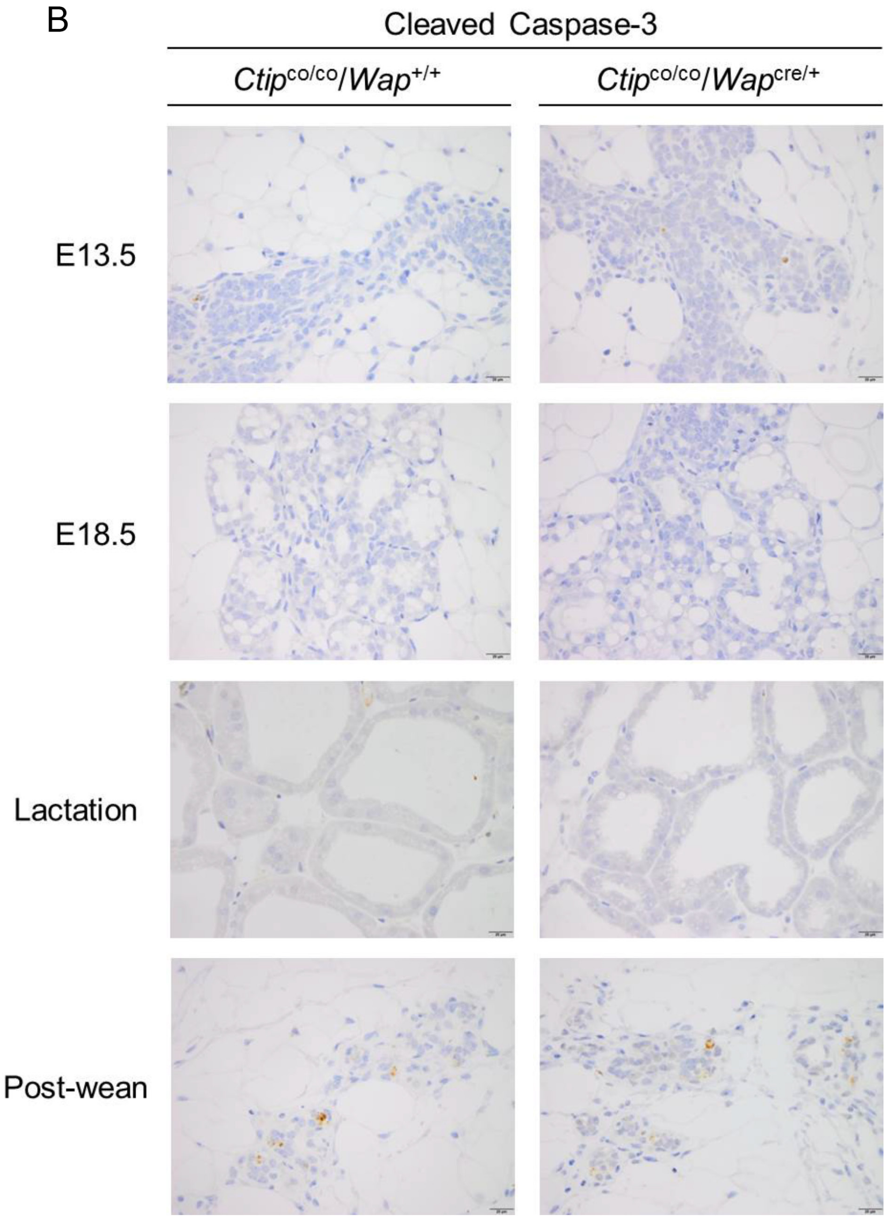

Supplementary Figure S2: Representative images of mammary glands stained with histochemical markers of cell proliferation (Ki67) or apoptosis (cleaved caspase-3). Sections of mammary gland #4 from pregnant (days E13.5 and E18.5), lactating (10 days postpartum), and weaned mother (10 days post-wean) mice were immunostained with antibodies for Ki67 (**A**) or cleaved caspase-3 (**B**). The proliferation and apoptosis indices presented in Figure 3B were calculated as the percentage, respectively, of Ki67- or caspase-3-positive luminal cells within the epithelial lining of tubuloalveolar structures.

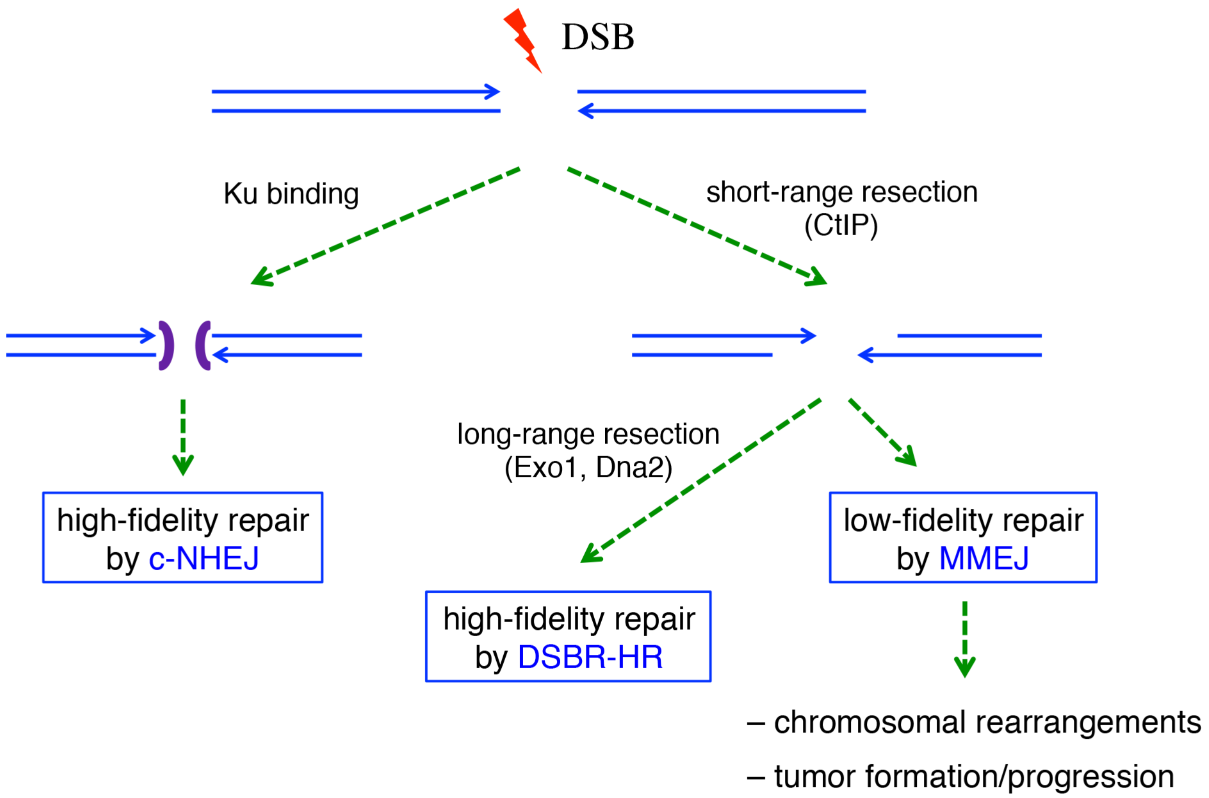

Supplementary Figure S3: A model to account for the effects of CtIP loss on mammary tumor formation. DNA double-strand breaks (DSBs) can potentially be repaired by either high-fidelity (c-NHEJ and DSBR-HR) or low-fidelity (MMEJ) pathways of DSB repair. Since the junctions of most chromosomal rearrangements of human breast tumors harbor sequence microhomologies [1], these abnormalities are likely to arise by MMEJ [2, 3], a repair pathway known to be dependent on CtIP. As such, loss of CtIP may inhibit mammary tumorigenesis by reducing MMEJ-dependent formation of oncogenic chromosomal abnormalities.

REFERENCES

1. Stephens PJ, McBride DJ, Lin ML, Varela I, Pleasance ED, Simpson JT, Stebbings LA, Leroy C, Edkins S, Mudie LJ, Greenman CD, Jia M, Latimer C, et al. Complex landscapes of somatic rearrangement in human breast cancer genomes. Nature. 2009; 462:1005–1010.
2. Zhang Y, Jasin M. An essential role for CtIP in chromosomal translocation formation through an alternative end-joining pathway. Nature Struct Mol Biol. 2011; 18:80–84.
3. Barton O, Naumann SC, Diemer-Biehs R, Kunzel J, Steinlage M, Conrad S, Makharashvili N, Wang J, Feng L, Lopez BS, Paull TT, Chen J, Jeggo PA, Lobrich M. Polo-like kinase 3 regulates CtIP during DNA double-strand break repair in G1. J Cell Biol. 2014; 206:877–894.
